# Learning from over ten years of implementing the One Health approach in the Democratic Republic of Congo: A qualitative study

**DOI:** 10.1016/j.onehlt.2024.100934

**Published:** 2024-11-15

**Authors:** Marc K. Yambayamba, Eric K. Kazadi, Belinda M. Ayumuna, Paulin M. Kapepula, Mathieu N. Kalemayi, Didier M. Kangudie, Justin Masumu, Boka O. Marcel, Serge T. Nzietchueng, Chloe Clifford Astbury, Tarra L. Penney, Nadège K. Ngombe, Simon R. Rüegg

**Affiliations:** a Section of Epidemiology, Vetsuisse Faculty, University of Zurich, Zurich, Switzerland; b Department of Epidemiology and Biostatistics, University of Kinshasa School of Public Health, Kinshasa, DR Congo; c Faculty of Veterinary Medicine, University of Kinshasa, Kinshasa, DR Congo; d Food and Agriculture Organization, Emergency Centre for Transboundary Animal Disease (ECTAD), DR Congo Country Office, Kinshasa, DR Congo; e Africa Field Epidemiology Network (AFENET), DR Congo Country Office, DR Congo; f Faculty of Pharmaceutical Sciences, University of Kinshasa, Kinshasa, DR Congo; g Commission de Coordination Une Santé de la République Démocratique du Congo, Ministère de l'Enseignement Supérieur et Universitaire, Kinshasa, DR Congo; h Breakthrough ACTION, Johns Hopkins University Centre for Communication Programs, DR Country office, DR Congo; i Institut National des Recherches Biomédicales (INRB), Kinshasa, DR Congo; j Université Pédagogique Nationale (UPN), Kinshasa, DR Congo; k Food and Agriculture Organization, Emergency Centre for Transboundary Animal Disease, Regional Office for Africa (RAF), Accra, Ghana; l Food and Agriculture Organization, Emergency Centre for Transboundary Animal Disease, Eastern and Southern Africa, Nairobi, Kenya; m Faculty of Veterinary Medicine, University of Liege, Belgium; n School of Global Health, York University, Toronto, ON, Canada; o Dahdaleh Institute for Global Health Research, York University, Toronto, ON, Canada

**Keywords:** One Health (OH) framework, National One Health platform, Integrated approaches to health, Decentralization, Global Health security, Health promotion

## Abstract

**Background:**

The Democratic Republic of Congo (DRC) has faced emerging infectious diseases such as Ebola, Mpox and Yellow fever, and antimicrobial resistance is a growing concern. To address these issues, in 2011 the country embarked on implementing the One Health (OH) approach at the national and provincial levels. This study investigates OH institutionalization and implementation in the DRC, describes the process of OH decentralization, and identifies the opportunities and challenges of sustaining these efforts.

**Methods:**

We conducted a qualitative study based on literature, document review and key informant interviews. The literature search targeted PubMed, Google Scholar and the document depository of the national One Health platform (NOHP). Key informant identified at the national level included ministry representatives, OH platform members and donors supporting OH implementation. These interviews were conducted in-person and online, recorded, transcribed, and imported into Dedoose software (version 9.2.006) for coding. Content analysis was performed to identify activities, processes, and achievements during the implementation of OH in DRC.

**Findings:**

Results of the literature and document review (*n* = 72) and analysis of stakeholder interviews (*n* = 24) indicate that a national OH platform, initiated in 2011, is hosted at the Ministry of Higher Education and coordinates other sectors. It comprises governmental departments, academic institutions, and civil society organizations working at the human, animal, and environment sectors. The governance structure includes a national coordinator, a permanent secretariat, technical working groups, and subnational entities at provincial and territorial levels. Following the establishment of the national OH platform, a structured process foresees to facilitate OH implementation at the provincial and territorial levels. Achievements up to today include the development of training programs, establishment of OH committees in some provinces, assessments of workforce needs, formulation of a national strategy, development of governance manuals, and support to the Mpox response coordination.

Nevertheless, OH implementation in the DRC faces challenges, including leadership tensions at the national level, inadequate domestic funding, limited training and capacity building for professionals, and insufficient infrastructure for data collection and sharing. Strengthening leadership and coordination, advocating for domestic resource mobilization, and strengthening infrastructure for data collection and sharing while ensuring equity across sectors is essential for advancing the OH agenda and ensuring its efficacy.

## Highlights

• One Health, an integrated approach, is advocated to strengthen health systems and disease prevention and management at the national and subnational levels.
• We present lessons learnt from One Health implementation in the Democratic Republic of Congo, which provide insights on best practice, challenges, and gaps.
• Sustainability will be achieved by mobilizing more domestic resources and training professionals. Mid-term, intersectoral tensions must be addressed, and infrastructures for data collection and sharing should be established to achieve the expected impact.

## 1 Introduction

Current health challenges have increased the call for multisectoral collaboration using integrated approaches to health such as One Health (OH) [[1], [2], [3]]. To address such challenges caused by emerging infectious diseases, climate change, land use change, or other complex issues, it has been noted that working in silos fails to achieve the expected impact [4,5]. The reason lies in the intertwined dependencies of people, animals, plants, and ecosystem wellbeing. At the global level, key organizations including the World Health Organization (WHO), the Food and Agriculture Organization of the United Nations (FAO), and the World Organization for Animal Health (WOAH) started promoting the intersectoral collaboration as part of the tripartite agreement [6]. They were joined recently by the United Nations Environmental Program (UNEP) to become the quadripartite, recognizing the importance of the environmental domain as highlighted by the OH High-Level Expert Panel (OHHLEP) OH definition [7]. The Tripartite developed and provided an operational tool aimed at guiding the establishment of multisectoral collaboration mechanisms at the country level [8,9]. Recently, a Quadripartite OH joint plan of action expressing the need to operationalize OH both at the national and sub-national levels was published, followed by the implementation guides [10,11]. The latter provide a five-step process for OH implementation, starting with a situation analysis of the existing multisectoral collaboration approaches [10].

In Africa, several countries have institutionalized OH by establishing national OH platforms at different governance levels with support from partners and OH networks [12]. However, the governance structure of these platforms tends to be unique to each country, reflecting country-specific organizational frameworks and priorities [13]. Progress has been made but challenges such as intersectoral tensions, limited resource mobilization, and limited workforce capacity continue to hinder the effective implementation of the OH approach [[14], [15], [16]].

The Democratic Republic of Congo (DRC) has been exposed to a high burden of zoonotic diseases, including 15 Ebola outbreaks since 1976, Mpox in the endemic form, and the recurrent Yellow fever, all in the context of a weak health system [[17], [18], [19], [20], [21], [22]]. Apart from these outbreaks, the country is facing a silent epidemic of antimicrobial resistance [23]. Key drivers such as poverty, armed conflicts, deforestation, food insecurity, a growing population, wild meat hunting, and low rural community preparedness contribute to occurrence of these diseases [1,[24], [25], [26], [27], [28], [29]]. Alongside these issues are the increasing burden of non-communicable diseases and endemic diseases such as Malaria, TB, and HIV which also require attention from health policy-makers [30]. Even though multidisciplinary response committees have been put in place during recent Ebola outbreaks, they did not endure at the end of the response. For more sustainable and better-coordinated preparedness and response activities, there is a need for an integrated approach to health at each level of governance [18,31]. Strengthening multisectoral collaboration was one of the main recommendations of the Joint External Evaluation of the International Health Regulations (JEE-IHR) conducted in 2018 in DRC [32]. A similar recommendation was made 5 years later during the second JEE-IHR conducted in 2023 (unpublished).

Even though efforts to implement OH started in the DRC more than 10 years ago, they are not documented in the scientific literature [33]. The purpose of this study is to examine the institutionalization and implementation of the OH approach at the national level and the process for provincial level implementation, while identifying challenges and opportunities for effective OH implementation.

## 2 Methodology

### 2.1 Study setting

The DRC is divided into 26 provinces led by governors, and Kinshasa is the capital city. The estimated human population in 2022 was one hundred million [34]. Provinces except Kinshasa, are divided into territories for a total of 145 territories overall led by territorial administrators. The country shares borders with Rwanda, Uganda, Tanzania, Zambia, Angola, Central Africa Republic, Republic of Congo, Sudan, and Burundi. In 2021, it was estimated that about 55.2 % of its territory is covered by equatorial forest, where rural populations and wildlife interact [32]. The DRC is experiencing instability in its Eastern region, resulting in food insecurity and lack of access to essential services including health services caused by violence, mobility restriction and resource availability [35]. By reducing access to health care service this instability impacted the response to the 2018–2019 Ebola outbreak [36,37].

### 2.2 Study design

This work is a qualitative study to describe OH implementation in the DRC from 2011 to 2023 using documents related to OH implementation supplemented by key informant interviews. OH implementation was conceptualized using the pillars for successful operationalization of OH proposed by Gwakisa et al. [38]: a) Institutionalization of the OH approach, b) decentralization or deconcentration of OH, c) achievements, d) funding OH implementation, e) challenges for OH implementation, and f) strategies to address challenges. OH governance is the institutional framework and processes established to foster multisectoral collaboration and achieve common goals regarding OH challenges [39]. The decentralization is the transfer of a central level responsibility to a subnational entity while the deconcentration, is transfer of power with supervision from the national level [40,41]. The NOHP employed these two strategies to transfer responsibility to provinces and territories while providing supervision and oversight.

The study was approved by the Kinshasa School of Public Health Ethical Review Board (ESP/CE/40/2022) and data collection was conducted from June 2023 to January 2024. Informed consent was obtained from each participant before the interview.

### 2.3 Data collection and participant selection and recruitment

#### 2.3.1 Identification of literature and policy documents

We used a systematic search strategy to identify peer-reviewed and grey literature. The following search string was used to retrieve published articles in the scientific literature: “One Health” AND “DR Congo” or “DRC” or “RD Congo” AND “Implementation“in PubMed and Google Scholar from 2010 to 2023 in December 2023. For governance, implementation and legal framework documents, a request was submitted to the national OH platform to provide access to documents regarding activity implementation since its inception. As the request was granted, a file with 526 documents was shared. The following inclusion criteria were applied for both literature and documents: (i) documents or articles describing the results of OH implementation including policy and implementation documents, (ii) activity reports and dissemination material in French or English were considered. Letters, terms of reference for activity implementation, budget for activity implementation were not included. After deduplication and assessment for suitability for the above-mentioned criteria 145 documents were reviewed of which 69 were included and from literature search out of 126 identified 3 were included for a total of 72 documents and articles (Appendix A).

#### 2.3.2 Document data extraction

An Excel spreadsheet was used to summarize the documents using the following key themes: document ownership, year of publication, document category (legal, governance, implementation documents and peer-reviewed papers), activity described, level of implementation, challenges identified during implementation, source of funding for activity implementation.

#### 2.3.3 Selection of interview participants

We identified interview participants through purposive sampling using the snowball method. The selection was based on the following criteria: (i) experience with OH implementation in DRC, (ii) research on OH-related topics, (iii) involvement in the national OH platform activities, and (iv) willingness and availability to participate in the study. An invitation to a semi-structured interview was sent by email to the potential identified participants meeting at least two criteria. The interviews were conducted in French from June 2023 to January 2024. Out of twenty-six invited, twenty-four participated in the interview.

#### 2.3.4 Interview data collection

Three trained data collectors with experience with qualitative data collection conducted key informant interviews. An interview guide (Appendix B) was developed based on Ndungu et al. [16]. The following topics were covered: the participant‘s experience with the OH approach including training, the level of involvement of their organization in OH implementation, key achievements, challenges for successful implementation and suggested solutions to these challenges. The interviews were conducted either face-to-face, using the online meeting platform or by phone depending on the interviewee‘s preference, and lasted between 20-30 minutes. They were recorded, and transcribed verbatim by two researchers experienced with qualitative data collection before data analysis. No personal information was collected from interviewees, transcriptions were coded using the participants' sectors and only the research team was able to access the data using a secure password.

### 2.4 Data analysis

To maintain accuracy, the transcripts underwent a consistency check against the original recordings by MY, a proficient French speaker. Data analysis started only after all the interviews were conducted and transcripts developed. We used a deductive content analysis approach [16], where key themes were identified prior to coding. These themes included: a) Institutionalization of the OH approach, b) decentralization of OH, c) achievements, d) source of funding for OH implementation, e) challenges for OH implementation, and f) strategies to address the challenges. These were informed by the pillars for successful operationalization of OH proposed by Gwakisa et al. [38]. The research team familiarized themselves with each transcript, divided the data into meaningful units, codes and categories which were subsequently linked to the themes, adjusting, and readjusting as needed [42]. The coding was initially conducted on one transcript and after agreement applied to the remaining transcripts. Dedoose version 9.2.006 was used to support coding [43]. All this process was maintained in French and quotes translated to English during the writing of the manuscript. Documents review data extracted into the Excel file was organized following the above-mentioned themes and summarized in a Word document. We triangulated document review and interview data to describe the legal framework, institutionalization, and operationalisation of OH. Emerging themes were triangulated between document review and interview data, and quotes used to highlight where needed. We used the consolidated criteria for reporting qualitative research (COREQ) checklist to report the study results [44].

## 3 Results

### 3.1 Description of the reviewed documents and study participants

A total of seventy-two (*n* = 72) documents related to OH implementation in DRC, three retrieved through the literature search and 69 from NOHP repository. More than half of them were implementation documents (Table 1).

**Table 1:** Reviewed documents (*n* = 72).

Categories	*N*	Documents
Legal framework	3	- Order of establishment of the OH platform in 2011. - Amended order of establishment of the OH platform in 2022. - Order of establishment of the OH federation.
Governance	10	- National OH Strategy 2022–2026 - Governance manuals - Internal rules - Decentralization guides - Rabies Elimination strategy 2022–2033 - National Action Plan for Health Security - RCCE strategic and operational plans - RCCE policy for the 6 priority zoonoses - DRC Integrated Disease Surveillance and Response 3rd Edition - Integrated Surveillance of Animal Disease and Response.
Implementation documents	56	- Activity reports - Workforce needs assessment - Semi-annual bulletin - Training modules
Peer reviewed papers	3	- Kambala et al., 2018 - Maltais et al., 2022 - Akilimali et al., 2023

The 24 interviewees represented a diverse spectrum of sectors and disciplines (Table 2), coming from Ministries of Health, Hygiene and Prevention; Livestock and Fisheries, Environment and Sustainable Development; Media and Communications, Interior, Security, and Decentralization, as well as Higher Education, and international organizations involved in the OH implementation in the DRC. Sixteen participants had more than five years of field experience in OH, and the majority mentioned having heard about the OH approach through training and capacity-building activities or conferences. Half of them were from the human health sector, eight from animal health, and three from the environment sector.

**Table 2:** Interview participants characteristics (*n* = 24).

Characteristics	*N*
Sectors	
Human Health	12
Animal Health	8
Environmental Health	3
Others	1

Affiliations	
Government	13
Academia	8
International organizations	3

Experience with OH	
O-2 years	4
3–5 years	4
More than 5 years	16

### 3.2 The OH framework in the DRC

The National OH platform (NOHP) was established on 9th November 2011 by an executive order issued by the Ministry of Higher Education where it is currently anchored [33]. The missions of the NOHP are, a) to promote the implementation of the OH approach in the country by raising awareness, b) to develop a national OH strategy, and c) to coordinate the implementation of OH activities. The member institutions are: the Ministry of Higher Education; Ministry of Agriculture; Ministry of Fisheries and Livestock; Ministry of Interior, Decentralization, Security and Customary Affairs; Ministry of Health, Hygiene and Prevention; Ministry of Primary, Secondary and Vocational Education; Ministry of Media and Communication; Ministry of Environment and Sustainable Development; two university institutions (University of Kinshasa School of Public Health and University of Lubumbashi School of Veterinary Medicine); and the One Health Federation. The One Health Federation was established in 2011 to foster collaboration between professional associations in charge of human health, animal health, and pharmacies. The organization is a member of the NOHP representing the views of practitioners. The executive order was revised in 2022 to further include the Ministry of Social Affairs, Humanitarian Action and National Solidarity, and the Ministry of Scientific Research and Technological Innovation. The revised executive order provided the mandate for subnational level implementation while clarifying governance structure and missions for these subnational entities [33].

#### 3.2.1 Governance of the NOHP

**Fig. 1:**
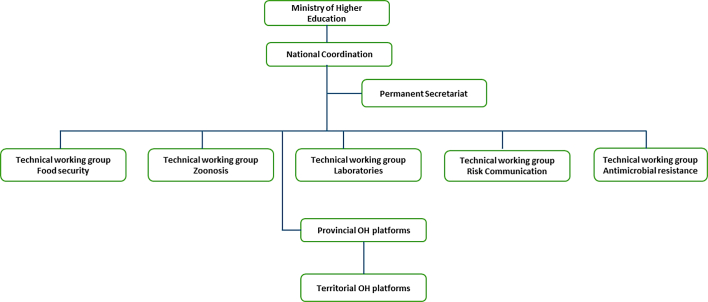
Organigram of the National One Health Platform of the DRC.

The NOHP governance manual describes three levels of organization: the NOHP coordination, the provincial OH coordination, and the territorial coordination (Fig. 1). The national coordinator provides leadership and coordination of OH implementation, the permanent secretariat provides administrative and financial support and dedicated technical working groups technical support at all levels of implementation. The NOHP is led by a coordinator from the Ministry of Higher Education while the permanent secretariat is made up of appointed representatives from the ministries of Health, Hygiene and Prevention; Environment and Sustainable Development; and Fisheries and Livestock [33]. There are five technical working groups dedicated to Zoonoses, Food security, Antimicrobial resistance (AMR), Laboratories, and Risk communication for community engagement (RCCE). The technical working groups provide more in-depth scientific and field insights into their respective topics.

#### 3.2.2 The national OH strategy

The national OH strategy (2022–2027) aims to strengthen multisectoral and multidisciplinary collaboration, coordination and cooperation in response to health priorities at the human-animal-environment interface using the OH approach with the following objectives: “ (i) effectively prevent, detect, and respond to health threats at the Human-Animal-Environment interface, (ii) establish a sustainable and institutionalized OH platform at all levels (national, provincial, and territorial), (iii) establish a strategic research agenda and sustained capacity for the implementation of OH initiatives (iv) intensely raise awareness of all stakeholders for the OH strategy, (v) strengthen the engagement with and support for OH by government and other stakeholder”.

#### 3.2.3 Governance of the sub-national One Health platform

The sub-national OH platforms' role is to implement the national strategy according to the local context. The governance structure of the subnational platforms is composed of the coordinator and an operational secretariat. Currently, OH is being implemented at the provincial level; and these provincial platforms will thereafter establish territorial OH platforms [45]. All their activities are accompanied by the national level.

### 3.3 Process of OH decentralization in DRC

**Fig. 2:**
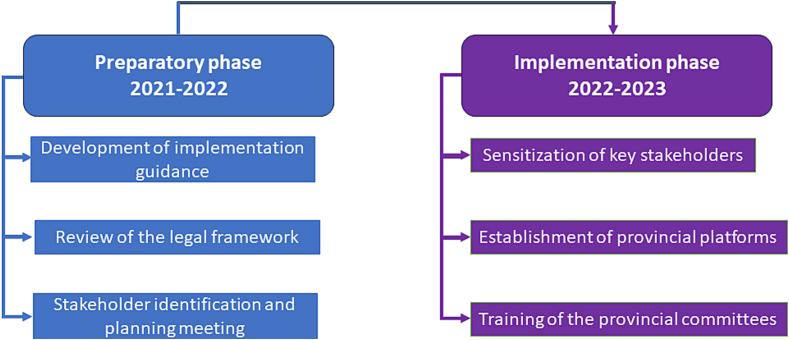
Process of provincial level decentralization of OH.

The decentralization of the OH platform in the DRC was planned in two phases with three steps each (Fig. 2), the preparatory phase for development and review of the legal framework, development of the governance manual, planning, and engagement with provincial level actors, and the implementation phase for the establishment of provincial-level platforms in selected provinces and training their members. All this process was led by the Ministry of Interior, Security, and Decentralization, which oversees local administration and engaged with governors through formal communication to introduce the activity.

#### 3.3.1 Decentralization preparatory phase

##### 3.3.1.1 Development of implementation guidance

The first step in the process to decentralize OH in the DRC was the development of key documents outlining the process and the governance of subnational entities. The NOHP held a one-week workshop in 2021 involving national level actors to develop and validate a governance manual and internal guiding rules for the subnational OH platforms. This twenty-page document provides a clear understanding of the platform governance, roles, and responsibilities of committee members [45]. It was followed by the technical guide for the decentralization of the OH platforms, which describes the process of implementing OH at the provincial and territorial levels, clarifying governance mechanisms, and selecting committee members at all levels.

##### 3.3.1.2 Review of the legal framework

The NOHP reviewed the legal framework and proposed and obtained an amended executive order from the Ministry of Higher Education. The updated document included the mandate to establish platforms in all 26 provinces and defined the governance model to be followed at the provincial and territorial levels following the above-mentioned guidance [45].

##### 3.3.1.3 Stakeholder identification and planning meetings

As a last step of the preparatory phase, and to get a clear understanding of the local context and identify who should be part of the provincial platform, planning meetings were organized between the national level and the key contacts at the provincial level. The provincial representatives were mostly heads of divisions working under the provincial ministries of public health, livestock, fisheries, and environmental and sustainable development, with representatives from the academia involved in OH-related research activities. The provincial participants conducted a mapping of key organizations and partners involved in OH activities and presented a summary of findings during the planning meetings. At least two to three planning meetings were organized with each province before the establishing the provincial platforms.

#### 3.3.2 Decentralization process implementation phase

##### 3.3.2.1 Sensitization of provincial stakeholders

Delegates from the NOHP held a two-day introductory workshop in each province to introduce OH to provincial stakeholders, explain the framework of the OH platform in DRC, and the mandate for the provincial OH platform. They aimed to provide a clear understanding of the national strategy and clarify the roles and responsibilities of the members of the provincial platform. These workshops were chaired by the governors of the provinces or their representatives, or one of the three OH-relevant ministries at the provincial level such as the ministers of Health, Hygiene and Prevention, Livestock and Fisheries, Environment and Sustainable Development. After these sessions, the governor of the province formally appointed members of the provincial OH platform according to the results of the stakeholder analysis in the preparatory phase.

##### 3.3.2.2 Establishment of subnational level OH platform

**Fig. 3:**
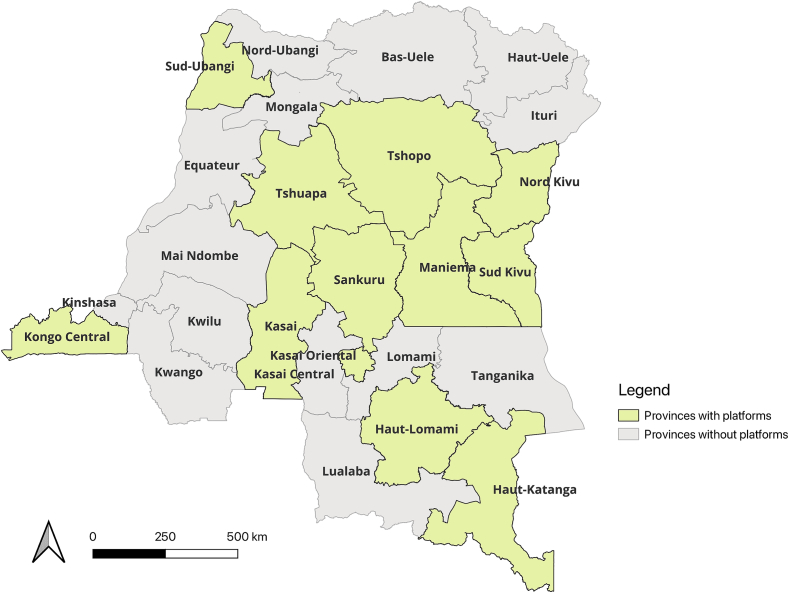
Map of the Democratic Republic of Congo highlighting provinces where provincial One Health platforms have been implemented by the national OH platform.

From June 2022 to July 2023, 12 out of twenty-six 26 provinces (46 %) established provincial OH committees using an executive order issued by the Governors of the provinces. These provinces are Haut-Katanga, Haut-Lomami, Kasai Central, Kasai-Oriental, Kongo Central, Maniema, Sankuru, Sud-Kivu, Sud-Ubangi, Nord-Kivu, Tshopo and Tshuapa (Fig. 3). The decentralization process is ongoing for the remaining provinces depending on funding availability. An additional provincial OH platform supported by WHO was established in the Equateur province not in the figure as the establishment process was not coordinated by the NOHP.

##### 3.3.2.3 Training of provincial committees

As a last step, a training module developed by the NOHP is being used to provide training to all the established provincial committees. This will provide a better understanding of OH to support their planning and activity implementation.

### 3.4 One Health implementation in DRC

#### 3.4.1 Key achievements

Interview opinions and documents review highlighted achievements of the NOHP from inception in 2011 to December 2023, these are summarized in Table 3.

**Table 3:** key achievements of the NOHP from 2011 to 2023.

Achievements	Year
*Training and capacity-building activities*: - Development and validation of OH training modules and materials for in-service professionals and pre-service students. - For in-service professionals: several residential training workshops were organized for key actors from different sectors. - Pre-service student short courses and Integration of OH competencies into the training curriculum in some universities.	2011–2023
*OH workforce needs assessment* - Workforce needs assessment conducted in 2018 including all the 7 ministries part of the NOHP. - Validation of the report highlighting gaps in human resource capacity by all the stakeholders.	2018
*Development of the National Strategy, governance manuals and dissemination tool* - The National OH strategy was developed and adopted by all the stakeholders for the period 2022–2026. - Governance manuals and internal regulations were developed and validated by all the members of the platform. - Publication of the quarterly One Health newsletter to disseminate information relating to One Heath activities.	2021
*Development of other strategies including*: - National integrated dog transmitted Rabies control plan 2022–2033. - National risk communication for community engagement (RCCE) strategy and operational plans. - National RCCE policy for the 6 priority zoonoses and standard operating procedures.	2021–2022
*Coordination of the Mpox response* - A Mpox response plan was developed and validated by key stakeholders. - Response activities such as case investigation, joint risk assessment (JRA), RCCE, training, and capacity building were coordinated by the platform.	2022–2023
*Sub-national level implementation of OH* - Development of operational plans and roadmaps for decentralization of OH. - Establishment of subnational level OH platforms in 12 provinces of the country.	2022–2023

These achievements contributed to the IHR core capacities human resources (C6), preparedness (C7), RCCE (C10) and, zoonosis (C12).

#### 3.4.2 Funding of OH activities

DRC government provides in-kind support for One Health activity implementation. Funding provided for One Health activities is donor dependent. Participants highlighted that USAID funding through the Global Health Security Agenda (GHSA) was instrumental in establishing the OH workforce capacity and implementing OH activities both at the national and provincial levels. This funding is provided through implementing partners such as the FAO-ECTAD, AFROHUN, and Breakthrough ACTION as mentioned by participants during interviews. AFROHUN is a network of universities and higher education institutions in 10 countries, including the DRC, focusing on training and capacity building of current and future OH professionals [46]. The FAO ECTAD DRC is implementing the Emergency Centre for Transboundary Animal Diseases (ECTAD) [47] which also provides training and institutional support. Breakthrough ACTION is supporting the country's risk communication and community engagement capacity strengthening using the OH approach. Breakthrough ACTION is implemented by Johns Hopkins University Centre for Communication Programs [48]. Working with FAO, WHO provided support during the development of the rabies elimination strategy [49]. An animal health interviewee described the situation as follows:

Currently [for activity implementation] we have to go through donors like FAO, like USAID, Breakthrough ACTION. We need partners in any case*.*

Animal health interviewee

#### 3.4.3 One Health workforce capacity development

The NOHP, with support from donors, provided training and capacity building for professionals at the national level and in selected provinces. Training modules have been developed and used to train both professionals and students in selected universities. Training provided to professionals is diverse, covering OH principles, field epidemiology (FETP and ISAVET), AMR, emergency management practices, priority zoonotic diseases prevention and control. Implementation of OH at the provincial level increases the need to expand these opportunities. Working with local government and donors can help to mobilize funding for this training.

For sustainable workforce capacity development, participants noted that engaging universities into curriculum review and integration of OH competencies may be an important way forward, as some institutions have already embarked into this process:

At the university level, a good number of faculties are involved in the OH approach, and with experience, their presence has changed enormously, and many courses have been influenced. The way I taught the pharmacology course before I became aware of the OH approach, is not the same because now I have even integrated notions related to the involvement of other non-pharmacist or non-doctor actors in for example the rational use of medicines.

University academic 2

### 3.5 Challenges for One Health implementation in the DRC at the national and sub-national levels

The following challenges to implementing OH were identified: Limited government ownership, cross-sector coordination, limited funding, limited training and capacity building opportunities, and limited infrastructures, and resources for sector-specific activities.

#### 3.5.1 Limited government commitment to the OH initiative

The OH approach is explicitly mentioned in the government 2021–2023 action plan [50] as a key strategy for cross-sector collaboration. Despite this high level recognition, interviewees mentioned limited government commitment as a key challenge for implementation of the OH strategy. To tackle this challenge, high level advocacy and awareness raising activities targeting senior government official were suggested.

At the [national] government level, the lack of a clear vision where it looks like the government is not convinced, because if the government was convinced, the support would be obvious and would be practical [...] the practical support of the State, this support is hesitant, it is not seen in a concrete way concerning several dimensions.

University academic 1

Secondly, it is the fact that most of our authorities have not yet been fully exposed to the OH approach. It's a huge challenge because you have to explain, raise awareness, and advocate so that people can understand the one health approach.

Member of the NOHP 1

#### 3.5.2 Intersectoral tensions

In the context of cross-sector collaboration and coordination, it is noteworthy that the NOHP, while encompassing members representing various key ministries, currently operates under the purview of the Ministry of Higher Education. Historically, this was seen as a way of resolving intersectoral tensions, particularly between the human health and animal health sectors. Consequently, there have been notable challenges in achieving full engagement from the Ministry of Health at the national level. During stakeholder interviews, a prevailing concern emerged, with over half of the participants underscoring that the Ministry of Higher Education may not possess the legitimacy to spearhead an organization that brings together diverse ministries. This question has given rise to tensions and has hindered some structures of the Ministry of Health, Hygiene and Prevention to engage with the NOHP.

The context of the OH approach as long as I can remember was not easy because it involved several sectors and so there was the problem of who coordinates this approach. Is it health or the environment or the Ministry of Fisheries, Livestock and Agriculture? There was this context undermined by this tension that was difficult to resolve. This is already the problem, and it persists even today. It's not clear.

Human health interviewee 2

…The first challenge is the leadership conflict. We need to overcome this problem by moving towards shared leadership.

Member of the NOHP 1

A solution to this issue proposed by participants was to move the NOHP from the Ministry of Higher Education to the Prime Minister's office. For them, this high-level leadership will bring everyone to the table and increase the government's awareness:

There is a need for a commission reporting to the authority above the ministries, such as the Prime Minister. I applaud the steps taken by the National Public Health Institute to ensure that we have a multisectoral framework within which we will have the different compartments, including epidemiological surveillance, laboratory, both animal and human care, environmental health management.

Human health interviewee 3

However, for some participants, doing this would reduce the platform's capacity to implement OH activities.

It's bringing it (the platform) to a neutral ground, yes… but here we risk falling into another problem, the administrative burden... It (the Prime Minister's office) takes care of many other issues, and we risk falling into another challenge of administrative burden [high workload and other pressing issues].

Academia 1

#### 3.5.3 Limited domestic funding of OH activities

Most of the participants highlighted limited domestic funding as a challenge for sustaining activities and reaching country-level coverage as these donors have specific objectives and do not cover all the country:

The first challenge is that the funding for OH activities is entirely provided by donors. So, if they withdraw their funding today, everything will fall apart. We have already started to support the OH platform to bring OH activities within the national budget. We shouldn't have national activities that are 100% dependent on external funding.

International organization interviewee 2

#### 3.5.4 Limited training and capacity building opportunities for OH

Training and capacity opportunities provided to date have not yet covered all the country's needs. This is exacerbated by the high turnover of government personnel at various levels. Participants highlighted that training and capacity building are key for a good understanding of the approach which can promote ownership.

We say, we install the administrators of the territories, they have training, we are sure that we have done very good training. A month later, we come back and say no, this administrator is no longer there, this administrator has been moved.

University academic 3

#### 3.5.5 Limited infrastructure for data collection and sharing

An integral aspect of the OH paradigm is information and sharing of data to facilitate multisectoral actions. Participants stated that data-sharing efforts led by the division of disease surveillance at the Ministry of Health is ongoing. Two challenges have been identified, firstly limited infrastructure and human resource capacity across the human, animal, and environmental sectors, and secondly the difference in terms of administrative organization. The human health system follows a 3-tier level organization (National, provincial and health zones) with the other sectors following the country administrative organization (National, provincial and territories). A territory can have more than one health zone. The animal health and environmental sectors are based at the territorial cities with at times long distances from health zones to these cities. The human health sector is well equipped in terms of staffing and data collection from the community to the health zone where data is encoded in the online routine surveillance systems, in contrast, the other sectors are under resourced.

There is now a data sharing framework, we meet at least once a month with the different sectors at the level of the Directorate-General for Disease Control to share information, but we ask ourselves the question does all the information from the other sectors get collected and reach the national level? We don't know, we can report information every week for the whole country, but they don't have that capacity yet.

Human health interviewee 4

The aim is to create a network at the level of national parks, a network of health actors, veterinarians who can at any time give information here at the headquarters concerning mortalities, diseases that are recorded in protected areas and then discuss from time to time, and see how we can intervene in the event of an epidemic, in the event of a disease that occurs in protected areas. We're still at the grassroots level organizing the program, so far it's not even organized yet, we're really at the beginning.

Environmental sector interviewee 1

## 4 Discussion

The NOHP has been established with high-level political support. The executive order of the creation was presented and approved by the government during a government cabinet meeting in 2011 and reviewed in 2022. The current institutional structure of the NOHP includes stakeholders such as civil society, professional bodies, universities, and media. This aligns with the One Health High Level Expert Panel (OHHLEP) understanding of OH and inclusion moving from traditional sectors to include the environment, wildlife, and conservation, as well as civil society [7]. The institutional anchor of the platform is at the Ministry of Higher Education. This has raised intersectoral tensions impacting the engagement of the Ministry of Health, especially the disease control division. In the African continent, several governance models and institutional mechanisms to establish the OH platform have been used, from presidential orders and prime ministerial decrees to memoranda of understanding between ministries. In Tanzania, the OH platform is hosted at the Prime Minister's office [52,53], while in Kenya, a memorandum of understanding between the Ministry of Health and the Ministry of Agriculture, Livestock and Fisheries established the Zoonotic Disease Unit acting as the OH platform, with ongoing discussions around including the environment [54]. There is no perfect structure or document for the establishment of OH platforms. Instead, this process needs to integrate the country's organizational culture, existing structures, and needs. Investing in OH governance is key for sustainable implementation both at the national and subnational level [13].

In DRC, efforts are ongoing to strengthen collaboration between the NOHP and the National Public health Institute. In December 2023, these two organizations attended a workshop on “Optimizing the Coordination of Public Health Emergency Response in Francophone Africa,” organized by the Agency for Preventive Medicine (AMP) in Abidjan. The aim of the workshop was to enhance coordination and collaboration in responding to health emergencies. A consensus between stakeholders is needed on the institutional mechanisms while securing the achievements and the technical capacity to deliver. Extensive evidence highlights that coordination challenges negatively impact the implementation of OH activities both at the country and continental level [[15], [16], [58], [59]].

Domestic resource mobilization is a key challenge for sustainability of OH activities and platforms [60]. In the African region, OH implementation has been technically and financially supported by donors under the Global Health Security Agenda with limited domestic resources [[15], [52], [54],^58^,^61^]. Despite the high political commitment to OH implementation globally, more resources are still allocated to the human health sector [62]. In Tanzania, Gwakisa et al. found that political commitment was key for optimizing OH interventions in complex settings [63]. In Kisumu County, Kenya, and Uganda, the lack of funding was identified as one of the key challenges for the implementation of OH activities [15,64]. Looking at the achievements, only two technical working groups (zoonosis and RCCE) in the DRC are operational. This may be driven by existing technical and financial support or limited prioritization of the remaining areas. There are two key issues with donor-funded implementation. First, the project-driven agenda means only activities falling into the donor‘s scope will be implemented [62]. Second, the sustainability of the subnational-level platforms beyond project funding may be challenging without national and local government funding [65,66]. Increasing domestic funding for OH activities require a high-level advocacy for political engagement coordinated by the platform. The diversity of the platform members provides an opportunity to explore other sources of funding, targeting The Pandemic Funds for example [67]. It should be mentioned that implementing such a complex and large system requires time and sustained investment with a long-time perspective in mind [68].

The decentralization of OH followed a structured process and benefited from political support from the Ministry of Interior, in charge of local administration. Half of the provinces have established provincial OH platforms with financial support from donors, leaving behind provinces without donors' support. Donors' alignment with the existing mechanism will certainly enhance institutionalization while promoting mutualization of resources and efforts, avoiding duplication [69]. Political commitment at the provincial level is another challenge for resources mobilization. High turnover of political leaders at various levels contributes to the slow implementation of the approach in the country. For OH platforms to endure both at the national and sub-national levels, committee members will need to continuously engage with decision-makers, political leaders and the public to raise awareness. As provincial platforms are being implemented, strengthened coordination and information sharing across sectors and between stakeholders are important. For provincial level OH platforms to meet the promise, there is a need to contextualize to local conditions, and design and implement context-specific OH interventions while providing better supervision [63].

Combining a document review and key informant interviews, this study provides an in-depth description of OH institutionalization while identifying challenges for sustainable implementation. Despite the valuable information, this study has some limitations. Documents as data source were retrieved from the NOHP repository, which may have led to omission of other key documents and program implementation reports available elsewhere. Interviews focused on actors based at the national level and their views may not represent those at the subnational levels. Achievements of the NOHP were collected using participants opinions and reviewed documents. No evaluation was conducted to measure the impact of these on OH implementation. There is only a small network of people involved in implementing OH in the DRC for the last ten years. In such a network few interviews such as 15 should be enough to attain saturation [70]. However, this small number may also convey a risk of bias. We acknowledge that our study may not fully represent the diversity of perspectives within this community.

## 5 Conclusion

The implementation of the OH approach in the DRC has seen considerable progress, yet several critical challenges persist. This study has examined the ongoing efforts to establish OH initiatives at various levels within the country and has identified key challenges that must be addressed to ensure the sustainability and effectiveness of these endeavours. Limited government commitment and domestic funding mobilized to implement the 2022–2026 OH strategy, and leadership tensions have emerged as impediments to the successful implementation of OH programs. Addressing them is imperative to foster cooperation and coordination among the various stakeholders involved in OH initiatives. Additionally, prioritizing capacity building and training for both the current and future workforce is crucial. Building a skilled and knowledgeable body of professionals is essential for the long-term success of OH efforts.

## Funding statement

This manuscript was supported by the Canadian International Development Research Centre through the DOPERAUS project (Nr 109812). TLP & CCA declare funding provided by the 10.13039/501100000024Canadian Institutes of Health Research, Grant number VR5-172686.

## CRediT authorship contribution statement

**Marc K. Yambayamba:** Writing – original draft, Project administration, Methodology, Investigation, Formal analysis, Data curation, Conceptualization. **Eric K. Kazadi:** Writing – review & editing. **Belinda M. Ayumuna:** Writing – review & editing. **Paulin M. Kapepula:** Writing – review & editing. **Mathieu N. Kalemayi:** Writing – review & editing. **Didier M. Kangudie:** Writing – review & editing. **Justin Masumu:** Writing – review & editing. **Boka O. Marcel:** Writing – review & editing. **Serge T. Nzietchueng:** Writing – review & editing. **Chloe Clifford Astbury:** Writing – review & editing. **Tarra L. Penney:** Writing – review & editing. **Nadège K. Ngombe:** Writing – review & editing. **Simon R. Rüegg:** Writing – review & editing, Writing – original draft, Supervision, Methodology, Funding acquisition, Conceptualization.

## Declaration of competing interest

The authors declare the following financial interests/personal relationships which may be considered as potential competing interests:

Simon R. Ruegg reports financial support was provided by Canadian International Development Research Centre. Tarra Penny and Chloe Clifford Astbury reports financial support was provided by Canadian Institutes of Health Research. Marc K. Yambayamba reports a relationship with Africa One Health University Network (AFROHUN) that includes: employment. If there are other authors, they declare that they have no known competing financial interests or personal relationships that could have appeared to influence the work reported in this paper.

## Figures and Tables

**Figure:**
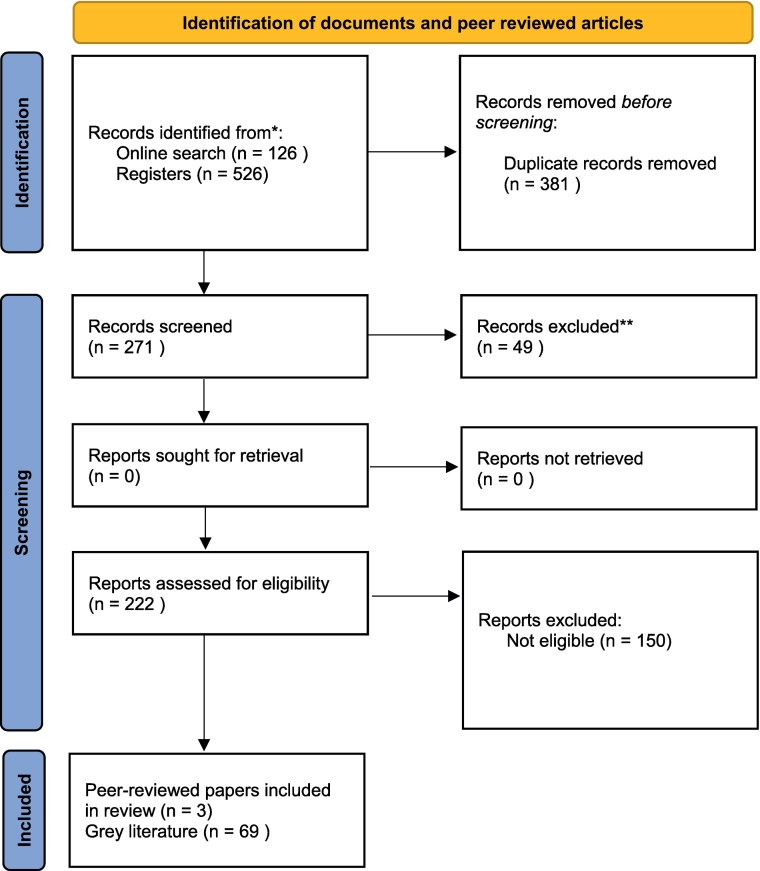


## Data Availability

Data will be made available on request.

## B. Interview guides

### Background

1. Which organization do you belong to?
2. In which department of this organization are you based?
3. What is your current position within your organization?
4. How long have you been with your current organization?
  a. One year or less
  b. 2–5 years
  c. 6–10 years
  d. More than 10 years

### Implementation and role

1. Have you ever heard of the One Health approach? On what occasion (year, place, and event)
2. To what extent is the One Health approach implemented in the DRC?
3. What role does your organization play in the implementation of this approach?
4. What are the barriers or challenges to implementing One Health approach in the DRC?
5. What are the key actions to promote One Health in the DRC?
6. Is there a framework for sharing surveillance data with other sectors? (Animal and Environmental Health) at what level is this framework implemented? How often are meetings held? When was the last meeting?
7. Who oversees the follow-up and implementation of the recommendations of these different meetings?
